# Colorimetric Method for Sensitive Detection of Microcystin-LR Using Surface Copper Nanoparticles of Polydopamine Nanosphere as Turn-On Probe

**DOI:** 10.3390/nano9030332

**Published:** 2019-03-02

**Authors:** Xiaodi Tang, Zhengzhi Yin, Xiaoling Lei, Yanbo Zeng, Zulei Zhang, Yixia Lu, Guobao Zhou, Lei Li, Xiaohua Wu

**Affiliations:** 1 College of Chemistry and Life Science, Zhejiang Normal University, Jinhua 321004, China; m18146683371@163.com; 2 College of Biological, Chemical Sciences and Engineering, Jiaxing University, Jiaxing 314001, China; 15757334248@163.com (X.L.); ybzeng@mail.zjxu.edu.cn (Y.Z.); jerry3641172@126.com (Z.Z.); luyixia@zjxu.edu.cn (Y.L.); gbzhou@mail.zjxu.edu.cn (G.Z.)

**Keywords:** turn-on visible absorption, dual-site recognition, magnetic separation, microcystin-LR, colorimetric sensor

## Abstract

A novel, facile sensor was further developed for microcystin-LR (MC-LR) determination by visible spectroscopy. Antibody-functionalized SiO_2_-coated magnetic nanoparticles (Fe_3_O_4_@SiO_2_) and aptamer-functionalized polydopamine nanospheres decorated with Cu nanoparticles (PDA/CuNPs) recognized specific sites in MC-LR and then the sandwich-type composites were separated magnetically. The Cu in the separated composites was converted to Cu^2+^ ions in solution and turn-on visible absorption was achieved after reaction with bis(cyclohexanone)oxaldihydrazone (BCO) (*λ*_max_ = 600 nm). There was a quantitative relationship between the spectral intensity and MC-LR concentration. In addition, under the optimum conditions, the sensor turns out to be a linear relationship from 0.05 to 25 nM, with a limit of detection of 0.05 nM (0.05 μg/L) (*S*/*N* = 3) for MC-LR. The sensitivity was dependent on the low background absorption from the off-to-on spectrum and label amplification by the polydopamine (PDA) surface. The sensor had high selectivity, which shows the importance of dual-site recognition by the aptamer and antibody and the highly specific color formed by BCO with Cu^2+^. The bioassay was complete within 150 min, which enabled quick determination. The sensor was successfully used with real spiked samples. These results suggest it has potential applications in visible detection and could be used to detect other microcystin analogs.

## 1. Introduction

Microcystins (MCs) are mainly formed as products of cyanobacterial metabolism. They are widely present in water. Structurally, MCs are monocyclic heptapeptides, that is, cyclo(d)-Ala-X-(d)-*erythro*-b-methyl-iso-Asp-Y-Adda-(d)-iso-Glu-*N*-methyldehydro-Ala and via peptide bonds, the seven amino acids are connected [1]. The unusual (2*S*,3*S*,8*S*,9*S*)-3-amino-9-methoxy-2,6,8-trimethyl-10-phenyldeca-4,6-dienoic acid (Adda) unit is unique to MCs. Besides it is associated with maternal toxicity and biological activity [2]. When leucine and arginine respectively occupied the positions, the corresponding MC-LR can not only strongly inhibit the bioactivities of phosphatases type 2A (PP2A) but also can inhibit type 1 (PP1), which have great importance in protein dephosphorylation. MC-LR is highly toxic even at low concentrations and is associated with most MC-induced toxic incidents [3,4]. The World Health Organization has already categorized MC-LR as a kind of neurotoxin and carcinogen. In addition, a maximum permissible limit of 1 μg/L has been established in drinking water [5]. Therefore, it is important to develop sensitive, specific, rapid and dependable methods for monitoring MC-LR.

In the past decade, more and more analytical techniques have been investigated for MC-LR detection. These methods, which include electroanalysis [6,7,8,9], photoelectrochemical [10,11,12,13], surface plasmon resonance [14], fluorescence [15,16] and colorimetric methods [3,17,18]. Colorimetric sensors are attracting increasing attention because of their simple construction, cost effectiveness, ease of use, potential for miniaturization and portability. Knopp’s group reported a competitive colorimetric method using MC-LR-functionalized magnetic beads and antibody-immobilized AuNPs, which could be stained for further detecting MC-LR [19]. Magnetic Fe_3_O_4_ is prominent for the rapid separation and other biocompatible nanoparticles also show potential application, such as CuNPs [20,21]. The signal source is important in achieving a good detection performance by a sensor and enzyme labels have been widely studied. However, the limited stability of enzyme labels restricts the applications of such sensors. There is therefore an urgent need to develop alternative enzyme-free labels that offer high reproducibility, ease of use, accuracy and portable detection to manage the health risks associated with MC-LR [22,23].

**Scheme 1:**
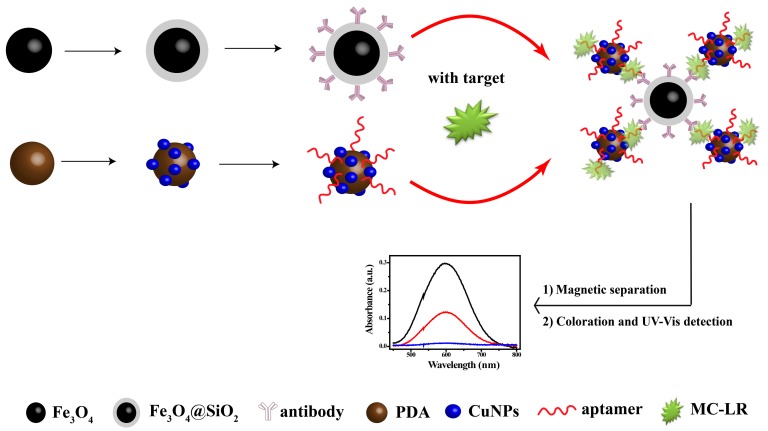
Schematic diagram of microcystin-LR (MC-LR) detection with sensor.

In this study, we used a relatively novel, convenient and efficient non-enzymatic strategy for detecting MC-LR (Scheme 1). MC-LR was captured via a dual recognition method by using an aptamer-coated Cu composite and antibody-coated magnetic nanospheres. The sandwich-type composite was then magnetically separated and the Cu was converted to Cu ions, which gave a turn-on ultraviolet-visible (UV-Vis) response with bis(cyclohexanone)oxaldihydrazone (BCO). The colorimetric complex provided a highly amplified and at the same time, it provided a reproducible signal for MC-LR analysis. In the detection process, MC-LR was captured in one step and it was also enriched using magnetic nanoparticles. Such particles provide a proper platform for eliminating pretreatment, with signal amplification and removal of matrix effects from many samples, leading to an off-to-on colorimetric complex. This straightforward assay enables effective detection with high sensitivity and specificity, with a performance that is better than those of previous analytical methods.

## 2. Materials and Methods

### 2.1. Materials

Dopamine hydrochloride, copper chloride, BCO, tetraethyl orthosilicate (TEOS), NaOH, tris(hydroxymethyl)aminomethane (Tris), poly(diallyldimethylammonium chloride) (PDDA, 20%, w/w in water, MW = 200,000–350,000), poly(acrylic acid) (PAA, 35%, w/w in water, MW = 100,000), 1-ethyl-3-(3-dimethylaminopropyl)carbodiimide hydrochloride (EDC), *N*-hydroxysuccinimide (NHS), ethanol and methanol were bought from the Aladdin Reagent Co., Ltd. (Shanghai, China). Tris–HCl buffer solution (0.2 M), phosphate–buffered saline (PBS, 0.2 M) and NH_3_–NH_4_Cl buffer solution (0.2 M) were used in the experiments. Milk, orange juice and drinking water were bought from a supermarket. River water was obtained from Jiaxing rivers. All these aqueous solutions were all prepared under the condition of doubly distilled water.

A monoclonal antibody (MC8C10) against MC-LR was bought from the Beijing Puhuashi Technology Development Co., Ltd. (located in Beijing, China); this antibody recognizes the Adda chain of the cyclic peptide. Microcystins (MC-LR and MC-RR), besides, okadaic acid (OA) were also bought from the corporation. Microcystins (MC-LF, MC-LW and MC-YR) were all purchased from Shanghai Enzo Life Sciences, Inc. As for Bovine serum albumin (BSA), it was purchased from the Beijing J & K Scientific Co., Ltd. (Beijing, China). The aptamer of MC-LR was synthesized by the Shanghai Sangon Biotechnology Co., Ltd. (located in Shanghai, China) and it was purified by HPLC; the sequence of it was 5′-NH_2_-GGC GCC AAA CAG GAC CAC CAT GAC AAT TAC CCA TAC CAC CTC ATT ATG CCC CAT CTC CGC-3′ [24]. Other chemicals, which were of analytical grade, were used without further purification and were purchased from the Shanghai Chemical Reagent Co. (Shanghai, China).

### 2.2. Equipment

UV-Vis absorption spectra were completely recorded with a UV2550 spectrophotometer. At the same time, transmission electron microscopy (TEM) was carried out with a Tecnai-G20 instrument (FEI, Hillsboro, Oregon, OR, USA). The magnetic behavior was investigated with a vibrating sample magnetometer (called Lake Shore 7410). Fourier-transform infrared (FTIR) spectroscopy was performed with a Nicolet Nexus-470 FTIR spectrometer (Thermo Scientific, Waltham, MA, USA). Thermal gravimetric analysis (TGA; STA-409PC, Netzsch Scientific Instruments, Ltd., Selb, Germany) and differential scanning calorimetry (DSC; 200PC, Netzsch Scientific Instruments, Ltd., Selb, Germany) were performed from ordinary room temperature to 800 °C and 450 °C, respectively (10 °C/min), in a nitrogen atmosphere.

### 2.3. Label Preparation

PDA nanospheres were prepared using a modified version of a previously reported procedure [25]. Ammonia aqueous solution (NH_4_OH, 1.0 mL, 28–30%) was mixed with ethanol (40 mL) and deionized water (90 mL) under the circumstance of mild stirring at room temperature for 30 min. Dopamine hydrochloride (0.5 g) was dissolved in deionized water (10 mL), then it was injected into the solution. The color of the mixture rightly changed to pale brown and then became dark brown. The whole reaction proceeded for 30 h. The PDA nanospheres were separated by centrifugation. After that, they were washed three times with water.

Cu nanoparticles (CuNPs) were prepared using a modified version of a previously reported method [26]. 800 μL of ammonia aqueous solution was added to copper chloride dihydrate solution (50 mL, 11.16 mmol) and at room temperature for 6 h under vigorous stirring. The products were purified by washing with water. Finally, CuNPs were obtained and dispersed in 20 mL water.

PDA/CuNP hybrids were prepared as follows [27]. PDA nanospheres were dispersed in an aqueous solution of 0.20% PDDA. And this solution contained 0.2 M Tris and 0.2 M NaCl. The dispersion was always stirred for 20 min for surface functionalization with PDDA. Residual PDDA can be removed by high-speed centrifugation and the complex was also rinsed with water at least three times. The composite (0.06 g) was dispersed in a CuNP solution (10 mL). What’s more, the mixture was stirred for 20 min. Centrifugation and further washing gave PDA/CuNPs.

PDA/CuNPs-aptamer labels were prepared as follows. PDA/CuNPs were dispersed under the above condition. And the dispersion was stirred for 30 min. The composite was separated by centrifugation and then it was washed with water to remove residual PDDA. It was dispersed under agitation in a solution containing 0.50% PAA for 30 min. Residual PAA was removed by high-speed centrifugation. The resulting composite was self-assembled with PDDA and PAA by the way of repeating the above steps. To activate the carboxyl groups of PAA, after surface modification, the obtained PDA/CuNPs were immersed in a solution containing EDC (1 mg/mL) and NHS (0.5 mg/mL) for 30 min. The hybrid was then immediately immersed in a mixture of 5 μM aptamer solution for 2 h at the temperature of 37 °C to yield biolabels (PDA/CuNPs-aptamer). The remaining active sites of PDA/CuNPs-aptamer were blocked by incubation in BSA (5%) for 2 h at 37 °C. The nanocomposite was separated and washed three times with Tris-HCl buffer (pH 7.4) and it was stored in pH 7.4 Tris-HCl buffer solution at 4 °C.

### 2.4. Preparation of Magnetic Probes

Fe_3_O_4_ nanospheres were synthesized by a solvothermal method that was modified [28]. FeCl_3_·6H_2_O (1.35 g) was dissolved in glycol (40 mL) to form a solution, followed by addition of sodium acetate (3.6 g) and poly(ethylene glycol) (1.0 g). After that, the mixture was stirred vigorously for 30 min and finally it was sealed in a Teflon-lined stainless-steel autoclave (50 mL capacity). This kind of autoclave was heated and maintained at 200 °C for 8 h and then it was cooled naturally. The product was collected with an external magnet and it was washed with ethanol and water.

Fe_3_O_4_@SiO_2_ nanoparticles were all prepared using a modified version of a previously reported method [29]. Fe_3_O_4_ (300 mg) was dispersed in a type of mixture of ethanol (40 mL) and water (4 mL). The mixture was sonicated for 15 min and ammonia (5 mL) and TEOS (2 mL) were gradually added under stirring. The series of reaction was performed for 12 h. In addition, the product was separated magnetically and it was washed three times with HCl (0.1 mol/L) and water and vacuum dried at the temperature 40 °C for 12 h.

A Fe_3_O_4_@SiO_2_@PAA nanocomposite was prepared using a previously reported procedure [30]. Fe_3_O_4_@SiO_2_ (1 g) was dispersed in dimethylformamide (DMF, 33 mL) and mixed with a solution of PAA (2 g) in DMF (33 mL). After sonication for 30 min, the mixture was heated to 110 °C under the condition of vigorous stirring. 4-Dimethylaminopyridine (0.033 g) dissolved in DMF (3.3 mL) and dicyclohexylcarbodiimide (0.33 g) dissolved in DMF (6.6 mL) were added dropwise. The mixture was stirred at 110 °C for 12 h. And the product was separated magnetically and washed three times with ethanol and water.

Fe_3_O_4_@SiO_2_-antibody immune probes were prepared by an established method [30]. Fe_3_O_4_@SiO_2_@PAA (6 mg) was dispersed in a solution that contained EDC (1 mg/mL) and NHS (0.5 mg/mL) for 30 min. After activation, the Fe_3_O_4_@SiO_2_@PAA was rinsed with Tris-HCl (pH 7.4) and immediately dispersed in an antibody solution (0.2 mg/mL) for 2 h to yield Fe_3_O_4_@SiO_2_-antibody. The composite was magnetically washed five times with Tris-HCl to remove residual antibodies. The remaining active sites of Fe_3_O_4_@SiO_2_@PAA-antibody were blocked by incubating with 5% BSA at 37 °C for 2 h. The composite was magnetically washed three with Tris-HCl. The probes were stored in Tris-HCl (5 mL, pH 7.4) at 4 °C before it was used.

### 2.5. Sensor Fabrication

Fe_3_O_4_@SiO_2_-antibody (20 μL, 0.6 mg/mL) and PDA/CuNPs-aptamer (15 μL, 12 mg/mL) were added to a solution containing an unknown concentration of MC-LR (65 μL). A schematic diagram of all the process can be found in Scheme 1. The mixture was shaken vigorously in a thermostatic shaker at the temperature of 37 °C for 2 h. Subjected the mixture to a magnetic field of a magnet for one minute and the non-magnetic constituents including unreacted biolabel were discarded in the process. The magnetically separated complexes were clearly washed three times with distilled water. Nitric acid (100 μL, 1.2 M) was added dropped to the composite (20 min) to dissolve the labeled CuNPs to give Cu ions. The solution pH was adjusted to weakly acidic with 1 M NaOH (110 μL). Sodium citrate solution (20 μL, 0.4 g/mL) and Tris-HCl (40 μL, pH 9.0) were added to the mixture. BCO (40 mL, 2 g/L) was added to the solution and reacted for 10 min. The colorimetric absorbance was used to determine the MC-LR concentration.

## 3. Results and Discussion

### 3.1. Characterization of Magnetic Beads and PDA/CuNP Hybrids

**Figure 1:**
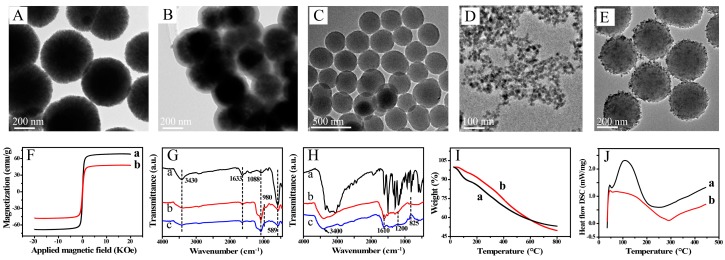
Transmission electron microscopy (TEM) images of Fe_3_O_4_ (**A**), Fe_3_O_4_@SiO_2_ (**B**), PDA (**C**), CuNPs (**D**) and PDA@CuNPs (**E**); (**F**) vibrating sample magnetometry curves for (a) Fe_3_O_4_ and (b) Fe_3_O_4_@SiO_2_; (**G**) Fourier transform infrared (FTIR) spectra of (a) Fe_3_O_4_, (b) Fe_3_O_4_@SiO_2_ and (c) Fe_3_O_4_@SiO_2_@PAA; (**H**) FTIR spectra of (a) dopamine hydrochloride, (b) PDA and (c) PDA@CuNPs; thermogravimetric analysis (TGA) (**I**) and differential scanning calorimetry (DSC) (**J**) curves for (a) PDA and (b) PDA@CuNPs.

TEM images of Fe_3_O_4_@SiO_2_ and Fe_3_O_4_ are shown in Figure 1. The figure shows spherical Fe_3_O_4_ particles of average diameter 200 nm, with a narrow size distribution. Fe_3_O_4_@SiO_2_ is quasi-spherical and wrapped in a layer of thickness 35 nm. The magnetic properties of Fe_3_O_4_ and Fe_3_O_4_@SiO_2_ were investigated (Figure 1F). The corresponding hysteresis loops show that the magnetic saturations were 67.8 and 47.9 emu/g, respectively. This indicates that the SiO_2_ shells decreased the magnetic strength. The FTIR spectra are shown Figure 1G. The characteristic peak at 589 cm^−1^ keeps in line with the Fe–O stretching vibration of Fe_3_O_4_ (Figure 1G,a). The characteristic peak at 1088 cm^−1^ is in line with the Si–O–Si stretching vibration. The peak at 980 cm^−1^ arises from Si–OH groups. In addition, the peaks at 3430 and 1630 cm^−1^ correspond to the O–H stretching and H–O–H bending vibration (Figure 1G,b and c). The results show that surface functionalization gave a flexible surface with functional groups and biocompatibility and adjusted the magnetic strength to give decreased bird-nesting; these effects are beneficial for bioapplications.

PDA is attracting increasing interest because of its special properties. The synthesized PDA nanospheres were uniformly spherical and of diameter ~300 nm (Figure 1C). The isoelectric point of PDA is at pH 4. In a weak alkali, the PDA spheres have a negative potential surface because of deprotonation of the phenol group, which can electrostatically adsorb cationic PDDA; a surface with homogeneously distributed positive charges is obtained. To enable rapid dissolution and a good sensing performance, CuNPs of diameter 8–12 nm were synthesized (Figure 1D). These negatively charged CuNPs were effectively anchored on the surfaces of the PDA nanospheres by electrostatic attraction (Figure 1E). The FTIR spectrum of the PDA nanospheres shows the characteristic peaks from several functional groups, for example, N–H, –OH, C–O and C–N (Figure 1H). The peak at 3400 cm^−1^ corresponds to O–H and N–H stretching. The peaks at 1610, 825 and 1200 cm^−1^ are attributed to the stretching vibrations of C=C, C–H and C–O, respectively. The TGA and DSC curves for PDA and PDA/CuNPs are shown in Figure 1I,J. The rate of weight loss for PDA/CuNPs was slower than that for PDA; the weight losses at 800 °C were approximately 50.28 and 46.81 wt%, respectively. The slower weight loss was mainly caused by the anchored CuNPs. The endothermal capacity decreased and the exothermal process was postponed by the introduction of CuNPs.

### 3.2. Proof of Concept for Proposed Sensor

**Figure 2:**
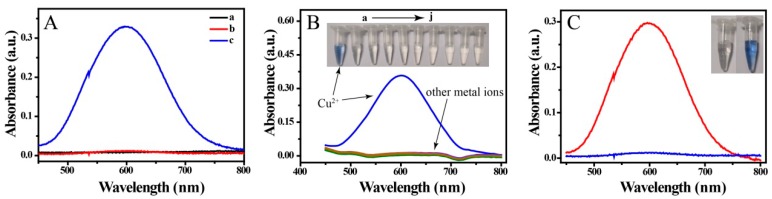
(**A**) Absorbance spectra of (a) Cu^2+^ and (b) bis(cyclohexanone)oxaldihydrazone (BCO) in solution of sodium citrate and triethanolamine and (c) mixture of Cu^2+^ and BCO in the solution; (**B**) absorbance spectra and photographs of BCO mixed with various metal ions, namely Cu^2+^, K^+^, Na^+^, Ag^+^, Mg^2+^, Ca^2+^, Cd^2+^, Co^2+^, Al^3+^ and Fe^3+^ (20 μM); and (**C**) absorbance spectra and photographs of sensing solution in absence (blue curve) and presence of MC-LR (15 nM, red curve).

Recently, off-to-on probes have attracted intense interest. As show in Figure 2A, Cu ions or BCO in a solution of sodium citrate and triethanolamine gave no UV-Vis absorption peak at 450 to 800 nm (Figure 2A,a and b). However, an intense absorbance was observed after mixing Cu^2+^ with BCO in solution, as a result of complex formation. The selectivity, which is important for metal ion probes, was explored by introducing various metal ions into a BCO solution, namely K^+^, Na^+^, Ag^+^, Mg^2+^, Ca^2+^, Cd^2+^, Co^2+^, Al^3+^ and Fe^3+^. As show in Figure 2B, coloration and colorimetric absorption were only observed in the presence of Cu^2+^; when other metal ions were added to BCO, the colorimetric absorption and solution color did not change compared with those of the substrate solution. These results indicate that the colorimetric probe has excellent selectivity and has high anti-interference when used in Cu-based analytical labels.

Here, Cu was used in a biolabel, as shown in Scheme 1. Proof of concept is necessary for a novel method. PDA/CuNPs-aptamer and Fe_3_O_4_@SiO_2_-antibody were added to a solution of MC-LR and incubated for 120 min. A magnet was then applied to the mixture and the non-magnetic components were discarded. Nitric acid was added and the pH was changed slightly to 9 Tris-HCl buffer solution and with NaOH. BCO was added and the mixture color changed to clear blue; the absorption spectrum λ_max_ was at 600 nm. This provided proof of concept and confirmed the feasibility of constructing a colorimetric sensor for MC-LR (Figure 2C). However, we observed a small peak at 600 nm for the immunosensor in the absence of MC-LR solution (Figure 2C). The detection conditions were therefore optimized as follows.

### 3.3. Optimization of Conditions

**Figure 3:**
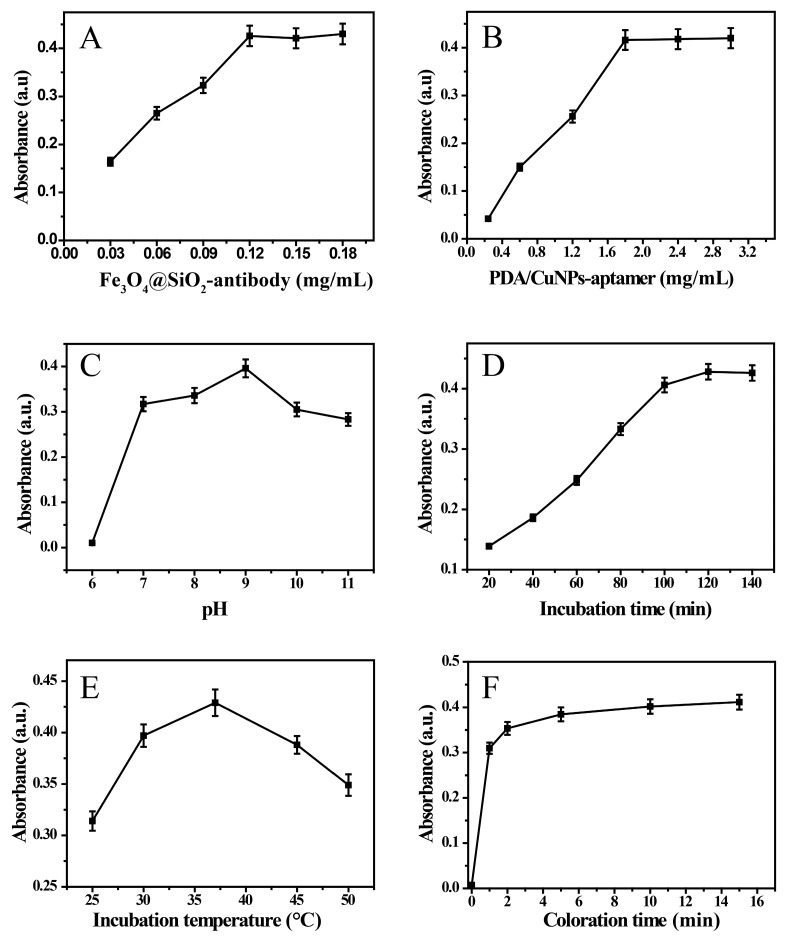
Effects of various concentration of Fe_3_O_4_@SiO_2_-antibody (**A**) and PDA/CuNPs-aptamer (**B**), pH (**C**), incubation time (**D**), incubation temperature (**E**) and coloration time (**F**) on signal intensity. MC-LR concentration was 25 nM. Error bars are standard deviations across three repeated experiments.

The concentrations of Fe_3_O_4_@SiO_2_-antibody and PDA/CuNPs-aptamer in analytic solution were first optimized to improve the MC-LR-sensing performance. Figure 3A shows that the absorbance intensities gradually increased with increasing Fe_3_O_4_@SiO_2_-antibody concentration (single variable) from 0.03 to 0.12 mg/mL and then became relatively stable; a similar trend was observed for PDA/CuNPs-aptamer (Figure 3B). These results indicate that the concentration of the label and magnetic probe were pivotal factors in the sensor performance. In subsequent experiments, Fe_3_O_4_@SiO_2_-antibody and PDA/CuNPs-aptamer concentrations of 0.12 and 1.8 mg/mL, respectively, were used. Next, the pH of the detection solution was optimized. The results show that the absorbance gradually increased with increasing pH from 6.0 to 9.0 and then it turned out to decrease at pH 9.0 to 11.0 (Figure 3C); this was related to the conformation and stability of the constituents. Subsequent experiments were performed at pH 9.0.

The specific interactions between molecules are affected by the reaction period and temperature. The final absorbance first increased with incubation time and then remained stable after 120 min (Figure 3D). To achieve high efficiency, 120 min was selected as the incubation time because a longer incubation time actually did not significantly increase the signal. The incubation temperature was also an important factor in the immunological reaction. Figure 3E shows that the maximum response signal was obtained after incubating at 37 °C. The signal intensity decreased at higher temperatures, which indicates that the biomolecular activity decreased at higher temperatures. The incubation temperature of 37 °C was therefore chosen in the process for the MC-LR sensor. The coloration time was also evaluated. As show in Figure 3F, the absorbance intensity increased sharply within the first 10 min and a stable absorbance was observed after 10 min. These results indicate that the reaction between Cu^2+^ and BCO is a fast-kinetic process. A coloration time of 10 min was used.

## 4. Analytical Performance of MC-LR Sensor

### 4.1. Calibration Curves

**Figure 4:**
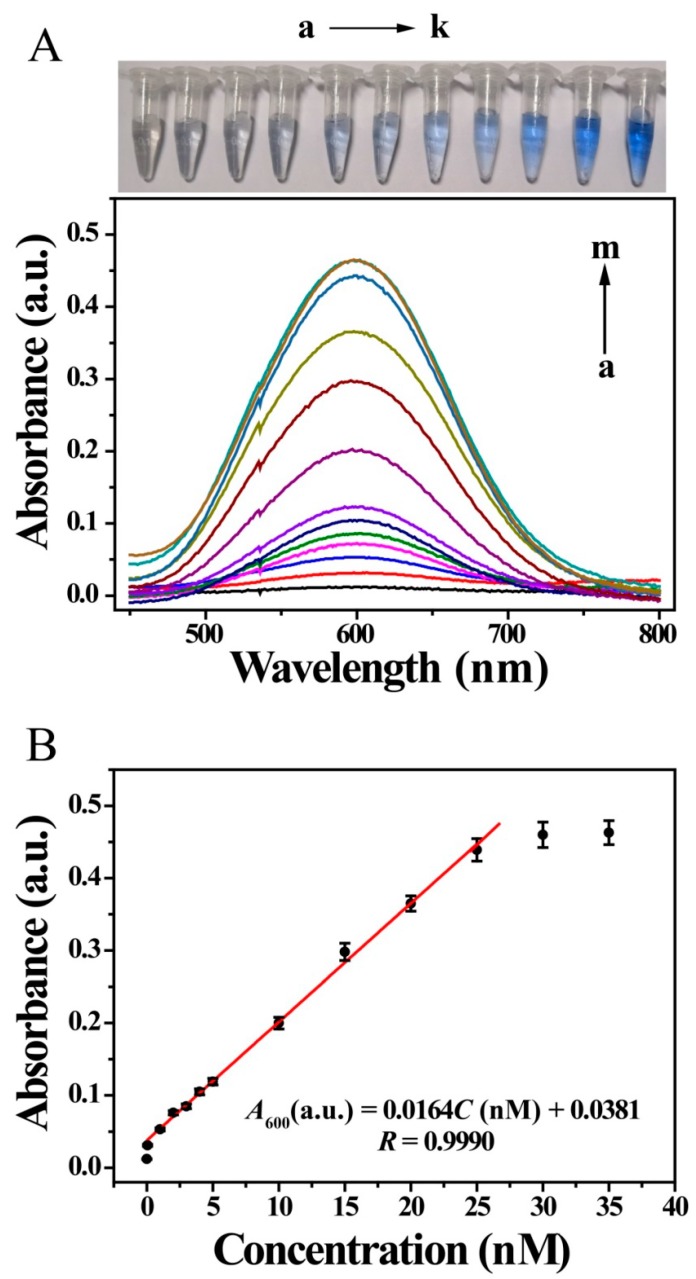
UV-Vis responses of sensor to various concentrations of MC-LR. (**A**) photographs of MC-LR solutions of various concentrations (a–k) and colorimetric spectra of sensor for various MC-LR concentrations (nM): (a) 0, (b) 0.05, (c) 1.0, (d) 2.0, (e) 3.0, (f) 4.0, (g) 5.0, (h) 10.0, (i) 15.0, (j) 20.0, (k) 25.0, (l) 30.0 and (m) 35.0; (**B**) corresponding calibration curve of absorbance values at 600 nm against MC-LR concentration.

Under optimized condition, the detection ability of the developed UV-Vis sensor was tested by using many different kinds of concentration of MC-LR. The photographs of the centrifuge tubes in Figure 4A show that as the MC-LR concentration increased, a distinct color change was observed: the color gradually changed from colorless to blue and then deeper blue. This indicates that the sensor has potential applications as a visual sensor. Further colorimetric results showed that the absorbance intensity at 600 nm tend to increase with increasing MC-LR concentration and a good linear relationship was obtained from 0.05 to 25 nM, with limit of detection (LOD) of 0.05 nM (0.05 μg/L) (*S*/*N* = 3) (Figure 4). The linear regression equation was *A*_600_ (a.u.) = 0.0164*C* [MC-LR] (nM) + 0.0381 (*R* = 0.9990). Under the condition that the concentration of MC-LR was higher than 25 nM, the absorbance tended to change slowly, and the relationship departed from linearity. The absorbance for 0 M MC-LR almost disappeared, compared with that in Figure 2C. Here, the background, including the non-specific signal, was negligible when considering signal enhancement in the presence of the target MC-LR, indicating an off-to-on process.

### 4.2. Selectivity, Reproducibility and Stability of MC-LR Sensor

**Figure 5:**
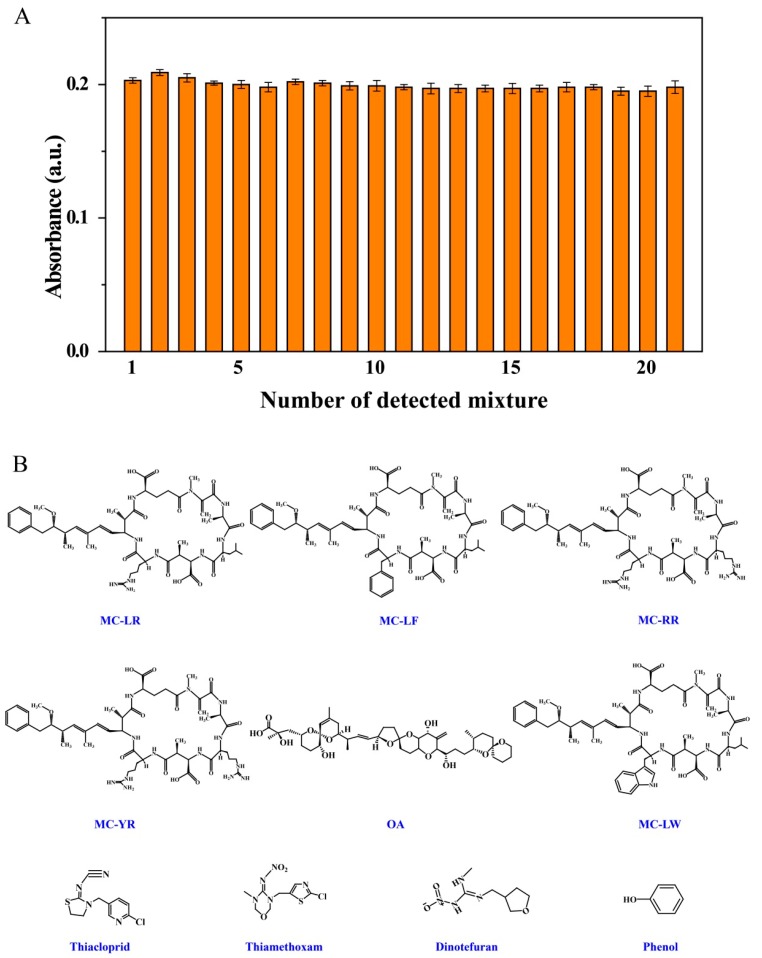
Effects of coexisting substances on colorimetric response of MC-LR. Solution composition: (**A**) (1) 10.0 nM MC-LR in Tris-HCl buffer solution (0.2 M, pH 7.4); mixtures of 10.0 nM MC-LR with (2) MC-LF, (3) MC-RR, (4) MC-YR, (5) OA, (6) MC-LW, (7) K^+^, (8) Cu^2+^, (9) Ag^+^, (10) Ca^2+^, (11) Na^+^, (12) Co^2+^, (13) Mg^2+^, (14) Al^3+^, (15) Fe^3+^, (16) Cd^2+^, (17) thiacloprid, (18) thiamethoxam, (19) dinotefuran and (20) phenol; (21) mixture of 1 with the other 20 types of interferent. (**B**) Molecular structures of interferents.

MCs are the most common cyanotoxins and have many variants. The selectivity of systems for their analysis is therefore important. The selectivity of the present colorimetric system was investigated by incubation with 10 nM MC-LR and other potential coexisting species, namely common cyanotoxins (100 nM, MC-RR, MC-LF, MC-LW, MC-YR and OA), metal ions (0.01 M, Na^+^, K^+^, Ag^+^, Ca^2+^, Mg^2+^, Cu^2+^, Co^2+^, Cd^2+^, Al^3+^ and Fe^3+^) and some organic compounds (0.01 M, phenol, acetamiprid, thiamethoxam, thiacloprid and dinotefuran). Figure 5A shows that interfering substances just had little influence on the sensing signals and the sensor displayed extremely high selectivity for MC-LR. The developed colorimetric sensor showed outstanding selectivity when used in the co-presence of other pollutants.

In fact, not only reproducibility but also stability is a very crucial factor in an analytical strategy. In order to evaluate the reducibility of the developed sensor, MC-LR (15 nM) detection was performed six times; the relative standard deviation (RSD), that is, 2.06%, indicated good reproducibility. The stability of the sensor was further evaluated as follows. After storage at 4 °C for 30 days, PDA/CuNPs-aptamer share a common analytical system in combination with newly prepared Fe_3_O_4_@SiO_2_-antibody; 99.3% of the initial response of PDA/CuNPs-aptamer to MC-LR was retained and it has high specificity. When Fe_3_O_4_@SiO_2_-antibody was stored at 4 °C for a month and then used in the analytical system with PDA/CuNPs-aptamer, 98.5% of the initial response of it to MC-LR was retained. When PDA/CuNPs-aptamer and Fe_3_O_4_@SiO_2_-antibody probes were used after storage at 4 °C for a month, the UV-Vis absorption intensity retained over 97.7% of the initial response to MC-LR. All these results indicate that the capturing components of the antibody-MC-LR-aptamer sandwich have good stability and specificity. These are due to the biocompatibility of the surface microenvironment in biomolecular conjugation.

### 4.3. Analytical Application of Fabricated Sensor

The accuracy and applicability of the proposed method were investigated by performing real sample analysis in real time. MC-LR was detected in spiked samples, namely commercial milk, orange juice, drinking water and river water. The data in Table 1 show that MC-LR recovery was 95.50–105.00% and the RSD was 1.34–3.10% for the spiked samples; these results imply that the sensor gives good precision and repeatability. The prepared sensor performance was therefore acceptable, and the sensor has potential practical applications in MC-LR detection in real samples.

### 4.4. Comparison of Analytical Performance

We studied the methods for MC-LR detection reported over the past decade; these include colorimetric analysis and fluorescence. The performance of the fabricated sensor was compared with the previously reported methods in terms of the LOD, linear interval, anti-interference ability. What’s more, recovery from real samples (Table 2). The results show that the LOD of the developed sensor was much lower than most of the previously reported values, indicating that PDA/CuNPs act as excellent biolabels in assay systems. Until now, the anti-interference ability of our sensor was the best, which suggests that the dual-recognition model is superior to other methods. In addition, the linear interval and recovery of our system were better than MC-LR that was previously reported. The comparison shows the significance of amplification by PDA/CuNPs nanocomposites in terms of the turn-on response.

## 5. Conclusions

In this study, a facile and efficient colorimetric strategy was developed for improved detection of MC-LR. PDA/CuNPs were used as biolabels and magnetic beads were used as separation probes. The special features of the sensor were the result of a dual-recognition strategy that involved an antibody-MC-LR-aptamer complex with low steric hindrance and specific color formation by interactions between Cu^2+^ and BCO. High sensitivity was achieved through the synergistic effect of releasable CuNPs and a low colorimetric background from off-to-on coloration. The magnetic probes provided a good platform for rapid enrichment and separation. Analysis could be accomplished within 150 min, with high selectivity, reproducibility and stability. This method performed well in real sample tests. The method has a wide range of potential applications in food security, clinical diagnosis and environmental monitoring by using different specific bioelements and could be used for visible detection.

## Figures and Tables

**Table 1:** MC-LR detection results obtained using proposed method (*n* = 6).

Samples	Added (nM)	Detected (nM)	RSD (%)	Recovery (%)
Drinking water	0	0	-	-
	4.00	4.05	2.10	101.25
	10.00	9.55	1.50	95.50
	20.00	19.12	2.33	95.60
Orange juice	0	0	-	-
	4.00	3.83	2.40	95.75
	10.00	10.37	3.10	103.70
	20.00	20.26	1.85	101.30
Milk	0	0	-	-
	4.00	3.95	1.36	98.75
	10.00	10.25	2.35	102.50
	20.00	20.89	2.76	104.45
River water	0	0	-	-
	4.00	4.20	1.34	105.00
	10.00	10.23	2.32	102.30
	20.00	19.57	1.84	97.85

**Table 2:** Comparison of analytical parameters for MC-LR detection obtained in this study and those reported for previous studies.

Detection Method	Labels	Linearity (nM)	LOD (nM)	Recovery (%)	Specificity	Reference
Fluorescence ^a^	Cy5.5	0.1–10.1	0.03	80–110	-	[31]
Fluorescence ^a^	Quantum dots 525	0.42–25	0.03	94–106.9	-	[32]
Fluorescence ^a^	Cy5	0.02–16	0.007	98.0–102.2	Highly	[33]
Fluorescence ^a^	Cy5.5	0.01–10	0.003	80–120	No significant	[34]
Fluorescence ^b^	FAM–labeled Apt	0.4–1200	0.138	88.46–112.1	Highly	[35]
Fluorescence ^a^	Cy5.5	0.36–2.5	0.21	84–108	-	[36]
Fluorescence ^a^	Europium nanospheres	0.1–5	0.035	94.6–102.8	-	[37]
Fluorescence ^b^	MoS_2_	0.01–50	0.002	94–112	Good	[15]
Fluorescence	DCHQ–Ph	0.1–10	0.05	98.8–118.9	-	[16]
Fluorescence Polarization ^a^	Fluorescein	-	0.86	88.6–118.8	-	[38]
UV–Vis ^a^	AuNPs/ hemin–CatG4	0.1–10	0.05	93.33–104.5	Highly	[17]
UV–Vis ^a^	AuNPs	0.51–11.68	0.38	88.8–117.5	-	[19]
UV–Vis ^b^	AuNPs	0.5–7.5 × 10^3^	0.37	98.5–102.2	Highly	[3]
UV–Vis ^b^	Methylene blue	0.65–1000	0.219	82.9–104.9	-	[18]
**UV–Vis ^a and b^**	**PDA/CuNPs**	**0.05**–**25**	**0.05**	**95.50**–**105.00**	**Excellent**	**This work**

**^a^** Antibody used as recognizing component. **^b^** Aptamer used as recognizing component. LOD: limit of detection. UV-Vis: UV-visible spectrophotometry. DCHQ-Ph: Phenyl-substituted diaza-18-crown-6 hydroxyquinoline.
